# Supplementary material for: A systematic scoping review to identify the design and assess the performance of devices for antenatal continuous fetal monitoring
Source: PLoS One. 2020 Dec 1;15(12):e0242983. doi: 10.1371/journal.pone.0242983 (PMC7707469; doi:10.1371/journal.pone.0242983)
Supplement: S1 Table — A detailed description of the electronic literature searches. (DOCX) [file pone.0242983.s001.docx]

# **S1 Table. Literature search history.**

**Database**: Ovid MEDLINE(R) 1946 to January, February 1 2020

**Date of search**: 12/02/2020

**Results**: 1300

| # | Search term | Results |
| --- | --- | --- |
| 1 | Prenatal Care/ | 26,837 |
| 2 | antepartum.ab.ti. | 5,174 |
| 3 | antenatal. ab.ti. | 29,802 |
| 4 | prenatal. ab.ti. | 82,864 |
| 5 | prepartum. ab.ti. | 2,041 |
| 6 | 1 or 2 or 3 or 4 or 5 | 128,288 |
| 7 | Fetal Monitoring/ | 7018 |
| 8 | ("continuous f?etal" adj5 monitor*).ab,ti. | 177 |
| 9 | ("continuous f?etal" adj5 devic*). ab,ti. | 3 |
| 10 | ("continuous f?etal" adj5 technolog*). ab,ti. | 0 |
| 11 | ("long-term f?etal" adj5 monitor*). ab,ti. | 5 |
| 12 | ("long-term f?etal" adj5 devic*). ab,ti. | 0 |
| 13 | ("long-term f?etal" adj5 technolog*). ab,ti. | 0 |
| 14 | "Monica AN24".ab,ti. | 10 |
| 15 | telefetalcare.ab,ti. | 1 |
| 16 | "f?etal magnetocardiography".ab,ti. | 102 |
| 17 | "f?etal vectorcardiography".ab,ti. | 1 |
| 18 | "f?etal ECG".ab,ti. | 361 |
| 19 | "f?etal electrocardiograph*".ab,ti. | 339 |
| 20 | "f?etal accelerometer*".ab,ti. | 0 |
| 21 | "accelerometer-based fetal activity monitor”.ab,ti. | 1 |
| 22 | "fetal movement accelerator monitor".ab,ti. | 0 |
| 23 | “actocardiograph*”.ab,ti. | 34 |
| 24 | 7 or 8 or 9 or 10 or 11 or 12 or 13 or 14 or 15 or 16 or 17 or 18 or 19 or 20 or 21 or 22 or 23 | 7626 |
| 25 | 6 and 24 | 1300 |

**Database**: EMBASE 1974 to 2020 week 5

**Date of search**: 12/02/2020

**Results**: 2112

| # | Search term | Results |
| --- | --- | --- |
| 1 | "PRENATAL CARE"/ | 37,862 |
| 2 | (antepartum).ti,ab | 7,588 |
| 3 | (antenatal).ti,ab | 48,007 |
| 4 | (prenatal).ti,ab | 115,456 |
| 5 | (prepartum).ti,ab | 2,333 |
| 6 | *(1 OR 2 OR 3 OR 4 OR 5)* | 179,566 |
| 7 | "FETUS MONITORING"/ | 10,779 |
| 8 | ("continuous f?etal" ADJ5 monitor*).ti,ab | 251 |
| 9 | ("continuous f?etal" ADJ5 devic*).ti,ab | 6 |
| 10 | ("continuous f?etal" ADJ5 technolog*).ti,ab | 1 |
| 11 | ("long-term f?etal" ADJ5 monitor*).ti,ab | 7 |
| 12 | ("long-term f?etal" ADJ5 devic*).ti,ab | 0 |
| 13 | ("long-term f?etal" ADJ5 technolog*).ti,ab | 0 |
| 14 | ("Monica AN24").ti,ab | 43 |
| 15 | (telefetalcare).ti,ab | 1 |
| 16 | ("f?etal magnetocardiography").ti,ab | 131 |
| 17 | ("f?etal vectorcardiography ").ti,ab | 2 |
| 18 | ("f?etal ECG ").ti,ab | 527 |
| 19 | ("f?etal electrocardiograph*").ti,ab | 254 |
| 20 | ("f?etal accelerometer*").ti,ab | 0 |
| 21 | ("accelerometer-based fetal activity monitor ").ti,ab | 1 |
| 22 | ("fetal movement accelerator monitor").ti,ab | 0 |
| 23 | (actocardiograph*).ti,ab | 42 |
| 24 | *(7 OR 8 OR 9 OR 10 OR 11 OR 12 OR 13 OR 14 OR 15 OR 16 OR 17 OR 18 OR 19 OR 20 OR 21 OR 22 OR 23)* | 11,474 |
| 25 | *(6 AND 24)* | 2,112 |

**Database**: CINAHL with full text EBSCOhost

**Date of search**: 12/02/2020

**Results**: 237

| # | Search term | Results |
| --- | --- | --- |
| 1 | "PRENATAL CARE"/ | 15,080 |
| 2 | (antepartum).ti,ab | 1,683 |
| 3 | (antenatal).ti,ab | 12,576 |
| 4 | (prenatal).ti,ab | 23,454 |
| 5 | (prepartum).ti,ab | 143 |
| 6 | *(1 OR 2 OR 3 OR 4 OR 5)* | 43,061 |
| 7 | "FETAL MONITORING"/ | 1,491 |
| 8 | ("continuous f?etal" ADJ5 monitor*).ti,ab | 4 |
| 9 | ("continuous f?etal" ADJ5 devic*).ti,ab | 0 |
| 10 | ("continuous f?etal" ADJ5 technolog*).ti,ab | 0 |
| 11 | ("long-term f?etal" ADJ5 monitor*).ti,ab | 0 |
| 12 | ("long-term f?etal" ADJ5 devic*).ti,ab | 0 |
| 13 | ("long-term f?etal" ADJ5 technolog*).ti,ab | 0 |
| 14 | ("Monica AN24").ti,ab | 7 |
| 15 | (telefetalcare).ti,ab | 0 |
| 16 | ("f?etal magnetocardiography").ti,ab | 0 |
| 17 | ("f?etal vectorcardiography ").ti,ab | 0 |
| 18 | ("f?etal ECG ").ti,ab | 3 |
| 19 | ("f?etal electrocardiograph*").ti,ab | 2 |
| 20 | ("f?etal accelerometer*").ti,ab | 0 |
| 21 | ("accelerometer-based fetal activity monitor ").ti,ab | 0 |
| 22 | ("fetal movement accelerator monitor").ti,ab | 0 |
| 23 | (actocardiograph*).ti,ab | 8 |
| 24 | *(7 OR 8 OR 9 OR 10 OR 11 OR 12 OR 13 OR 14 OR 15 OR 16 OR 17 OR 18 OR 19 OR 20 OR 21 OR 22 OR 23)* | 1,508 |
| 25 | *(6 AND 24)* | 237 |

**Database**: EMCARE

**Date of search**: 12/02/2020

**Results**: 756

| # | Search term | Results |
| --- | --- | --- |
| 1 | "PRENATAL CARE"/ | 15,257 |
| 2 | (antepartum).ti,ab | 1,989 |
| 3 | (antenatal).ti,ab | 15,223 |
| 4 | (prenatal).ti,ab | 26,878 |
| 5 | (prepartum).ti,ab | 178 |
| 6 | *(1 OR 2 OR 3 OR 4 OR 5)* | 46,180 |
| 7 | "FETUS MONITORING"/ | 3,412 |
| 8 | ("continuous f?etal" ADJ5 monitor*).ti,ab | 63 |
| 9 | ("continuous f?etal" ADJ5 devic*).ti,ab | 0 |
| 10 | ("continuous f?etal" ADJ5 technolog*).ti,ab | 1 |
| 11 | ("long-term f?etal" ADJ5 monitor*).ti,ab | 3 |
| 12 | ("long-term f?etal" ADJ5 devic*).ti,ab | 0 |
| 13 | ("long-term f?etal" ADJ5 technolog*).ti,ab | 0 |
| 14 | ("Monica AN24").ti,ab | 5 |
| 15 | (telefetalcare).ti,ab | 0 |
| 16 | ("f?etal magnetocardiography").ti,ab | 47 |
| 17 | ("f?etal vectorcardiography ").ti,ab | 0 |
| 18 | ("f?etal ECG ").ti,ab | 127 |
| 19 | ("f?etal electrocardiograph*").ti,ab | 67 |
| 20 | ("f?etal accelerometer*").ti,ab | 0 |
| 21 | ("accelerometer-based fetal activity monitor ").ti,ab | 0 |
| 22 | ("fetal movement accelerator monitor").ti,ab | 0 |
| 23 | (actocardiograph*).ti,ab | 11 |
| 24 | *(7 OR 8 OR 9 OR 10 OR 11 OR 12 OR 13 OR 14 OR 15 OR 16 OR 17 OR 18 OR 19 OR 20 OR 21 OR 22 OR 23)* | 3,588 |
| 25 | *(6 AND 24)* | 756 |

**Database**: BNI

**Date of search**: 12/02/2020

**Results**: 8

| # | Search term | Results |
| --- | --- | --- |
| 1 | "PRENATAL CARE"/ | 3,796 |
| 2 | (antepartum).ti,ab | 386 |
| 3 | (antenatal).ti,ab | 4,517 |
| 4 | (prenatal).ti,ab | 3,948 |
| 5 | (prepartum).ti,ab | 31 |
| 6 | *(1 OR 2 OR 3 OR 4 OR 5)* | 10,025 |
| 7 | ("continuous f?etal" ADJ5 monitor*).ti,ab | 19 |
| 8 | ("continuous f?etal" ADJ5 devic*).ti,ab | 0 |
| 9 | ("continuous f?etal" ADJ5 technolog*).ti,ab | 0 |
| 10 | ("long-term f?etal" ADJ5 monitor*).ti,ab | 0 |
| 11 | ("long-term f?etal" ADJ5 devic*).ti,ab | 0 |
| 12 | ("long-term f?etal" ADJ5 technolog*).ti,ab | 0 |
| 13 | ("Monica AN24").ti,ab | 0 |
| 14 | (telefetalcare).ti,ab | 0 |
| 15 | ("f?etal magnetocardiography").ti,ab | 1 |
| 16 | ("f?etal vectorcardiography ").ti,ab | 0 |
| 17 | ("f?etal ECG ").ti,ab | 9 |
| 18 | ("f?etal electrocardiograph*").ti,ab | 18 |
| 19 | ("f?etal accelerometer*").ti,ab | 0 |
| 20 | ("accelerometer-based fetal activity monitor ").ti,ab | 0 |
| 21 | ("fetal movement accelerator monitor").ti,ab | 0 |
| 22 | (actocardiograph*).ti,ab | 0 |
| 23 | *(7 OR 8 OR 9 OR 10 OR 11 OR 12 OR 13 OR 14 OR 15 OR 16 OR 17 OR 18 OR 19 OR 20 OR 21 OR 22)* | 44 |
| 24 | *(6 AND 3)* | 8 |

**Database**: Cochrane Library

**Date of search**: 12/02/2020

**Results**: 274 ((Cochrane reviews (21), Trials (253)).

| # | Search term | Results |
| --- | --- | --- |
| 1 | (“prenatal care”):ti,ab.kw | 2887 |
| 2 | MeSH descriptor: [Prenatal Care] this term only | 1395 |
| 3 | (“antepartum”):ti,ab.kw | 528 |
| 4 | (“antenatal”):ti,ab.kw | 4287 |
| 5 | (“prepartum”):ti,ab.kw | 69 |
| 6 | #1 OR #2 OR #3 OR #4 OR #5 | 6526 |
| 7 | (“fetal monitoring”):ti,ab.kw | 1545 |
| 8 | (“foetal monitoring”):ti,ab.kw | 1544 |
| 9 | MeSH descriptor: [Fetal Monitoring] this term only | 252 |
| 10 | #7 OR #8 OR #9 | 1545 |
| 11 | #6 AND #10 | 274 |

**Database**: Web of Science (Indexes=SCI-EXPANDED, SSCI, A&HCI, CPCI-S, CPCI-SSH, BKCI-S, BKCI-SSH, ESCI, CCR-EXPANDED, IC Timespan=All years)

**Date of search**: 12/02/2020

**Results**: 17

| # | Search term | Results |
| --- | --- | --- |
| 1 | TI=(prenatal OR antepartum OR antenatal OR prepartum) | 57,877 |
| 2 | TI=("continuous fetal monitor*" OR "continuous foetal monitor*") | 25 |
| 3 | #1 AND #2 | 1 |
| 4 | ALL=(prenatal OR antepartum OR antenatal OR prepartum) | 151,481 |
| 5 | ALL=("continuous fetal monitor*" OR "continuous foetal monitor*") | 72 |
| 6 | #4 AND #5 | 17 |

**Database**: Pubmed

**Date of search**: 12/02/2020

**Results**: 161

| # | Search term | Results |
| --- | --- | --- |
| 1 | ((((("prenatal care"[MeSH Terms] OR ("prenatal"[All Fields] AND "care"[All Fields]) OR "prenatal care"[All Fields]) OR antepartum[All Fields]) OR antenatal[All Fields]) OR prenatal[All Fields]) OR prepartum[All Fields]) AND ((((((((("foetal monitoring"[All Fields] OR "fetal monitoring"[MeSH Terms] OR ("fetal"[All Fields] AND "monitoring"[All Fields]) OR "fetal monitoring"[All Fields]) OR (Monica[All Fields] AND AN24[All Fields])) OR (("fetus"[MeSH Terms] OR "fetus"[All Fields] OR "fetal"[All Fields]) AND ("magnetocardiography"[MeSH Terms] OR "magnetocardiography"[All Fields]))) OR (("fetus"[MeSH Terms] OR "fetus"[All Fields] OR "fetal"[All Fields]) AND ("vectorcardiography"[MeSH Terms] OR "vectorcardiography"[All Fields]))) AND (("fetus"[MeSH Terms] OR "fetus"[All Fields] OR "fetal"[All Fields]) AND ("electrocardiography"[MeSH Terms] OR "electrocardiography"[All Fields] OR "ecg"[All Fields]))) OR (("fetus"[MeSH Terms] OR "fetus"[All Fields] OR "fetal"[All Fields]) AND ("electrocardiography"[MeSH Terms] OR "electrocardiography"[All Fields] OR "electrocardiograph"[All Fields]))) OR (("fetus"[MeSH Terms] OR "fetus"[All Fields] OR "fetal"[All Fields]) AND accelerometer[All Fields])) AND actocardiograph[All Fields]) OR ((continuous[All Fields] AND ("fetus"[MeSH Terms] OR "fetus"[All Fields] OR "fetal"[All Fields]) AND ("monitoring, physiologic"[MeSH Terms] OR ("monitoring"[All Fields] AND "physiologic"[All Fields]) OR "physiologic monitoring"[All Fields] OR "monitor"[All Fields])) OR (continuous[All Fields] AND ("fetus"[MeSH Terms] OR "fetus"[All Fields] OR "foetal"[All Fields]) AND ("monitoring, physiologic"[MeSH Terms] OR ("monitoring"[All Fields] AND "physiologic"[All Fields]) OR "physiologic monitoring"[All Fields] OR "monitor"[All Fields])))) | 161 |
