# Supplementary material for: A systematic scoping review to identify the design and assess the performance of devices for antenatal continuous fetal monitoring
Source: PLoS One. 2020 Dec 1;15(12):e0242983. doi: 10.1371/journal.pone.0242983 (PMC7707469; doi:10.1371/journal.pone.0242983)
Supplement: S2 Table — A list of the 63 articles that were excluded after full-text assessment and the reason for exclusion. (DOCX) [file pone.0242983.s002.docx]

# **S2 Table. Articles excluded after full test assessment.**

|  | Study citation details | Reason |
| --- | --- | --- |
| 1 | Ammala P and Kariniemi V. Short-term variability of fetal heart rate in cholestasis of pregnancy. Am J Obstet Gynecol. 1981; 141(2): 217-220. | Intermittent FHR trace (only done weekly) |
| 2 | American College of Obstetricians and Gynecologists. Practice bulletin no. 145: Antepartum fetal surveillance. Obstet Gynecol. 2014; 124(1): 182-192. | No relevant material |
| 3 | Avci R, Wilson JD, Escalona-Vargas D et al. Tracking Fetal Movement Through Source Localization From Multisensor Magnetocardiographic Recordings. IEEE J Biomed Health Inform. 2018; 22(3): 758-765. | No relevant material  (fMCG is not portable) |
| 4 | Blum T, Saling E and Bauer R. [Fetal magnetoencephalography I: 1st prenatal registration of auditory evoked neuromagnetic fields]. EEG-EMG Zeitschrift fur Elektroenzephalographie Elektromyographie und Verwandte Gebiete. 1984; 15(1): 34-37. | Unable to retrieve full text |
| 5 | Bracero LA, Roshanfekr D and Byrne DW. Analysis of antepartum fetal heart rate tracing by physician and computer. J Matern Fetal Med. 2000; 9(3): 181-185. | No relevant material  (uses CTG) |
| 6 | Brown R, Johnstone ED, Heazell AEP et al. Continuous objective recording of fetal heart rate and fetal movements could reliably identify fetal compromise, which could reduce stillbirth rates by facilitating timely management. Med Hypotheses. 2014; 83(3): 410-417. | Review article |
| 7 | Bureev AS, Zhdanov DS, Kiseleva EY et al. Comparative assessment of 24-hour fetal monitoring methods based on cardiac rhythm. Biosciences Biotechnology Research Asia. 2015; 12(2): 1743-1750. | Review article |
| 8 | Bushberg JT and Tupin JP Jr. RF Safety Analysis of a Novel Ultra-wideband Fetal Monitoring System. Health Phys. 2017; 112(5): 478-485. | No relevant material |
| 9 | Butler Tobah YS, LeBlanc A, Branda M et al. OB nest-a novel approach to prenatal care. Obstet Gynecol. 2016; 127: 7S‐8S. | Conference abstract |
| 10 | Casale MB, Pantelopoulos AA, Zurcher MA et al. Assessing the clinical use of a novel, mobile fetal monitoring device. Obstet Gynecol. 2014; 123(5): 55S. | No relevant material  (uses CTG) |
| 11 | Cerutti S, Civardi S, Bianchi A et al. Spectral analysis of antepartum heart rate variability. Clin Phys Physiol Meas. 1989; 10 Suppl B: 27-31. | No relevant material  (uses CTG) |
| 12 | Chang J and Caughey A. Inpatient, continuous fetal monitoring versus outpatient, antenatal tests in monoamniotic twins: a cost-effectiveness analysis. Am J Obstet Gynecol. 2008; 199(6): S201-S201. | Conference abstract |
| 13 | Chourasia VS, Gangopadhyay R, Tiwari AK et al. Foetal phonocardiographic signal denoising based on non-negative matrix factorization. J Med Eng Technol. 2012; 36(1): 57-66. | No relevant material |
| 14 | Chourasia VS and Tiwari AK. Design methodology of a new wavelet basis function for fetal phonocardiographic signals. Thescientificworldjournal. 2013; 2013: 505840. | No relevant material |
| 15 | Comani S, Alleva G, Di Luzio et al. Fetal magnetocardiographic mapping using independent component analysis. Physiol Meas. 2004; 25(6): 1459-1472. | No relevant material  (fMCG is not portable) |
| 16 | Comani S, Di Luzio S, Romani GL et al. Automatic detection of cardiac waves on fetal magnetocardiographic signals. Physiol Meas. 2005; 26(4): 459-475. | No relevant material  (fMCG is not portable) |
| 17 | Comani S, Di Luzio S, Romani GL et al. Characterization of fetal arrhythmias by means of fetal magnetocardiography in three cases of difficult ultrasonographic imaging. Pacing Clin Electrophysiol. 2004; 27(12): 1647-1655. | No relevant material  (fMCG is not portable) |
| 18 | Comani S, Srinivasan V, Alleva G et al. Entropy-based automated classification of independent components separated from fMCG. Phys Med Biol. 2007; 52(5): N87-97. | No relevant material  (fMCG is not portable) |
| 19 | Dawes GS, Moulden M and Redman CWG. System 8000: Computerized antenatal FHR analysis. J Perinat Med. 1991; 19(1): 47-51. | No relevant material  (uses CTG) |
| 20 | De Araujo DB, Barros AK, Estombelo-Montesco et al. Fetal source extraction from magnetocardiographic recordings by dependent component analysis. Phys Med Biol. 2005; 50(19): 4457-4464. | No relevant material  (fMCG is not portable) |
| 21 | De Lathauwer L, De Moor B and Vandewalle J. Fetal electrocardiogram extraction by blind source subspace separation. IEEE Trans Biomed Eng. 2000; 47(5): 567-572. | No relevant material |
| 22 | Freeman RK. The evolution of antepartum fetal testing methods. Am J Obstet Gynecol. 2003; 189(1): 310. | Letter to editor |
| 23 | Fujimoto Y, Honda N, Tojo R et al. Prenatal diagnosis of long QT syndrome by non-invasive fetal electrocardiography. J Obstet Gynaecol Res. 2009; 35(3): 555-561. | Case report |
| 24 | Fukushima A, Matsumoto A, Terata M et al. Prenatal diagnosis of fetal arrhythmias and assessment of autonomic nervous system activity by fetal magnetocardiograph. Open Med Dev J. 2012; 4: 13-21. | No relevant material  (fMCG is not portable) |
| 25 | Gough NA, Dawson AJ and Tomkins TJ. Antepartum fetal heart rate recording and subsequent fast transmission by a distributed microprocessor-based dedicated system. Int J Biomed Comp. 1986; 18(1): 61-65. | No relevant material (uses Doppler) |
| 26 | Govindan RB, Vairavan S, Ulusar UD et al. A novel approach to track fetal movement using multi-sensor magnetocardiographic recordings. Ann Biomed Eng. 2011; 39(3): 964-972. | No relevant material  (fMCG is not portable) |
| 27 | Hawrylyshyn PA, Organ LW and Bernstein A. A new computer technique for continuous measurement of the pre-ejection period in the human fetus: physiologic significance of pre-ejection period patterns. Am J Obstet Gynecol. 1980; 137(7): 801-809. | No relevant material (uses Doppler) |
| 28 | Hod M and Kerner R. Telemedicine for antenatal surveillance of high-risk pregnancies with ambulatory and home fetal heart rate monitoring--an update. J Perinat Med. 2003; 31(3): 195-200. | Review article |
| 29 | Hosono T. [Fetal magnetocardiography]. Rinsho Byori - Japanese Journal of Clinical Pathology. 2006; 54(5): 477-485. | Unable to retrieve full text |
| 30 | Kahler C, Grimm B, Schleussner E et al. The application of fetal magnetocardiography (FMCG) to investigate fetal arrhythmias and congenital heart defects (CHD). Prenat Diagn. 2001; 21(3): 176-182. | No relevant material  (fMCG is not portable) |
| 31 | Karikari Y, Siddiqui S and Javed Z. Fetal magnetocardiography: an essential tool in the pre and postnatal management of LQTS. Pediatr Cardiol. 2019; 40(1): 241. | Conference abstract |
| 32 | Khandoker A, Ibrahim E, Oshio S et al. Antepartum non-invasive evaluation of opening and closing timings of the cardiac valves in fetal cardiac cycle. Med Biol Eng Comput. 2009; 47(10): 1075-1082. | No relevant material  (uses Doppler) |
| 33 | Larks SD, Webster A and Larks GG. Quantitative studies in fetal electrocardiography. I. Prenatal prediction of the condition of the infant at birth (Apgar rating). Am J Obstet Gynecol. 1967; 98(1): 52-55. | No relevant material |
| 34 | Lewis MJ. Review of electromagnetic source investigations of the fetal heart. Med Eng Phys. 2003; 25(10): 801-810. | Review article |
| 35 | Li Y and Gonik B. Continuous fetal heart rate monitoring in patients with preterm premature rupture of membranes undergoing expectant management. J Matern Fetal Neonatal Med. 2009; 22(7): 589-592. | No relevant material (uses CTG) |
| 36 | Lowery C, Perissl H, Wilson JD et al. Neue perspektiven in der intrauterinen uberwachung mittels fetalem magnetenzephalogramm. Zentralblatt fur Gynakologie. 2003; 125(6): 218-225. | No relevant material  (fMCG is not portable) |
| 37 | Lowery C, Campbell JQ, Wilson JD et al. Noninvasive antepartum recording of fetal S-T segment with a newly developed 151-channel magnetic sensor system. Am J Obstet Gynecol. 2003; 188(6): 1491-1497. | No relevant material  (fMCG is not portable) |
| 38 | Mantini D, Alleva G and Comani S. A method for the automatic reconstruction of fetal cardiac signals from magnetocardiographic recordings. Phys Med Biol. 2005; 50(20): 4763-4781. | No relevant material  (fMCG is not portable) |
| 39 | Marchon N, Naik G and Pai KR. Linear Phase Sharp Transition BPF to Detect Noninvasive Maternal and Fetal Heart Rate. J Healthc Eng. 2018; 2018: 5485728. | No relevant material |
| 40 | Menendez T, Archenbach S, Moshage W et al. [Prenatal recording of fetal heart action with magnetocardiography]. Zeitschrift fur Kardiologie. 1998; 87(2): 111-118. | No relevant material  (fMCG is not portable) |
| 41 | Michel E, Brandt A, Laqua D et al. Non-invasive monitoring of fetal oxygen saturation." Klinische Padiatrie. 2010; 222. | Conference abstract |
| 42 | Mochimaru F, Fujimoto Y and Ishikawa Y. The fetal electrocardiogram by independent component analysis and wavelets. Jpn J Physiol. 2004; 54(5): 457-463. | No relevant material |
| 43 | Monincx WM, Zondervan HA, Birnie E et al. High risk pregnancy monitored antenatally at home. Eur J Obstet Gynecol Reprod Biol. 1997; 75(2): 147‐153. | No relevant material (uses Doppler) |
| 44 | Obata-Yasuoka M, Hamada H, Yoshikawa H et al. Clinical usefulness of foetal magnetocardiography. BJOG. 2012; 119. | Conference abstract |
| 45 | Petrikovsky BM, Sichinava L and Swancoat S. Can Fetal Heart Rate Responses to Maternal Exercise Be Used for Antepartum Surveillance? J Diagn Med Sonogr. 2018; 34(5): 342-345. | No relevant material (uses Doppler) |
| 46 | Quartero HW, Stinstra JG, Golbach EG et al. Clinical implications of fetal magnetocardiography. Ultrasound Obstet Gynecol. 2002; 20(2): 142-153. | No relevant material  (fMCG is not portable) |
| 47 | Quinn A, Weir A, Bain R et al. Antenatal fetal magnetocardiography: A new method for fetal surveillance? Br J Obstet Gynaecol. 1994; 101(10): 866-870. | No relevant material  (fMCG is not portable) |
| 48 | Quinn KH, Cao CT, Lacoursiere DY et al. Monoamniotic twin pregnancy: continuous inpatient electronic fetal monitoring-an impossible goal? Am J Obstet Gynecol. 2011; 204(2): 161.e161-166. | No relevant material  (electronic fetal monitoring) |
| 49 | Rassi D and Lewis MJ. Power spectral analysis of the foetal magnetocardiogram. Physiological Measurement. 1995; 16(2): 111-120. | No relevant material  (fMCG is not portable) |
| 50 | Salomon LJ, Mahieu-Caputo D, Jouannic JM et al. Fetal home monitoring for the prenatal management of gastroschisis. Acta Obstet Gynecol Scand. 2004; 83(11): 1061-1064. | No relevant material (uses Doppler) |
| 51 | Schmidt A, Witte R, Swiderski L et al. Advanced automatic detection of fetal body movements from multichannel magnetocardiographic signals. Physiol Meas. 2019; 40(8): 085005. | No relevant material  (fMCG is not portable) |
| 52 | Schramm K, Lapert F, Nees J et al. Acceptance of a new non-invasive fetal monitoring system and attitude for telemedicine approaches in obstetrics: a case-control study. Arch Gynecol Obstet. 2018; 298(6): 1085-1093. | No relevant material |
| 53 | Solum T, Ingemarsson I and Nygren A. The accuracy of abdominal ECG for fetal electronic monitoring. J Perinat Med. 1980; 8(3): 142-149. | No relevant material  (patients were intrapartum) |
| 54 | Stampalija T, Casati D, Maggi V et al. Analysis of fetal ECG in fetal growth restriction. Reprod Sci. 2014; 21(3). | Conference abstract |
| 55 | Sutha P and Jayanthi VE. Fetal Electrocardiogram Extraction and Analysis Using Adaptive Noise Cancellation and Wavelet Transformation Techniques. J Med Syst. 2017; 42(1): 21. | No relevant material |
| 56 | Van Leeuwen P, Schussler M, Bettermann H et al. [Magnetocardiography for assessment of fetal heart actions]. Geburtshilfe Frauenheilkd. 1995; 55(11): 642-646. | No relevant material  (fMCG is not portable) |
| 57 | Van Wijngaarden WJ, James DK and Symonds EM. The fetal electrocardiogram. Baillieres Clin Obstet Gynaecol. 1996; 10(2): 273-294. | Review article |
| 58 | Verdurmen KMJ, Lempersz C, van Laar JOEH et al. Normal ranges for fetal electrocardiogram values for the healthy fetus of 18-24 weeks of gestation: A prospective cohort study. BMC Pregnancy Childbirth. 2016; 16(1). | No relevant material |
| 59 | Wakai RT, Wang M, Pedron Sl et al. Spectral analysis of antepartum fetal heart rate variability from fetal magnetocardiogram recordings. Early Hum Dev. 1993; 35(1): 15-24. | No relevant material  (fMCG is not portable) |
| 60 | Wilson JD, Govindan RB, Hatton JO et al. Integrated approach for fetal QRS detection. IEEE Trans Biomed Eng. 2008; 55(9): 2190-2197. | No relevant material  (fMCG is not portable) |
| 61 | Yuan L, Yuan Y, Zhou Z et al. A Fetal ECG Monitoring System Based on the Android Smartphone. Sensors (Basel). 2019; 19(3). | No relevant material |
| 62 | YumotoY, Satoh S, Koga T et al. Prenatal diagnosis of slow-rate ventricular tachycardia using fetal electrocardiography. Prenat Diagn. 2004; 24(6): 463-467. | Case report |
| 63 | Zhao H, Chen M, Van Veen BD et al. Simultaneous fetal magnetocardiography and ultrasound/Doppler imaging. IEEE Trans Biomed Eng. 2007; 54(6 Pt 2): 1167-1171. | No relevant material  (fMCG is not portable) |
